# Quantification of Rare Circulating Tumor Cells in Non-Small Cell Lung Cancer by Ligand-Targeted PCR

**DOI:** 10.1371/journal.pone.0080458

**Published:** 2013-12-06

**Authors:** Jiatao Lou, Suqin Ben, Guohua Yang, Xiaohui Liang, Xiaoqian Wang, Songshi Ni, Baohui Han

**Affiliations:** 1 Department of Clinical Laboratory, Shanghai Chest Hospital Affiliated to Shanghai Jiao Tong University, Shanghai, China; 2 Department of Pulmonary Medicine, Shanghai Chest Hospital Affiliated to Shanghai Jiao Tong University, Shanghai, China; 3 Department of Respiratory Medicine, Affiliated Hospital of Nantong University, Nantong, Jiangsu, China; 4 Department of Research and Development, GenoSaber Biotech Co. Ltd., Shanghai, China; 5 Department of Respiratory Disease, the First People's Hospital Affiliated to Shanghai Jiao Tong University, Shanghai, China; Univesity of Texas Southwestern Medical Center at Dallas, United States of America

## Abstract

**Background:**

Quantification of circulating tumor cells (CTC) is valuable for evaluation of non-small cell lung cancer (NSCLC). The sensitivity of current methods constrains their use to detect rare CTCs in early stage. Here we evaluate a novel method, ligand-targeted polymerase chain reaction (LT-PCR), that can detect rare CTCs in NSCLC patients.

**Methods:**

CTCs were enriched by immunomagnetic depletion of leukocytes and then labeled by a conjugate of a tumor-specific ligand and an oligonucleotide. After washing off free conjugates, the bound conjugates were stripped from CTCs and then analyzed by qPCR. To evaluate the clinical utility, blood samples were obtained from 72 NSCLC patients (33 initially diagnosed and 39 on chemotherapy), 20 benign patients, and 24 healthy donors.

**Results:**

Experiments with healthy blood spiked with tumor cells indicated the LT-PCR allows specific detection of CTC. The clinical study showed that the initially diagnosed patients have an average of 20.8 CTC units with metastatic diseases, 11.8 CTC units with localized diseases, and 6.0 CTC units with benign diseases. With the threshold of 8.5 CTC units, the assay can detect 80% of stage I/II, 67% of stage III, and 93% of stage IV cancer. With the benign patients and healthy donors as control group, the method can detect cancer with a sensitivity of 81.8% and a specificity of 93.2%.

**Conclusion:**

The LT-PCR would allow quantification of CTC in NSCLC patients at a more sensitive level, providing a potential tool for stratifying malignant lung diseases, especially at early stage.

## Introduction

Circulating tumor cells (CTC), known as “liquid biopsy”, is a readily accessible marker for monitoring cancer progression, response to therapy and recurrence of malignant diseases. Especially, CTC becomes even more important when tissue biopsy is not suitable for certain patient population. Clinical significance of CTC in NSCLC has been widely reported in recent studies. High numbers of detected CTC were reported to associate with poor prognosis in metastatic lung cancer [1]–[5]. Detection of certain mRNA or multi-gene in CTC can be used for prognosis of the outcome of debulking surgery and radiotherapy in NSCLC patients [6]–[10]. More recently, molecular characterization of CTC in NCSLC has been shown to potentially guide therapy [1], [11].

**Figure 1:**
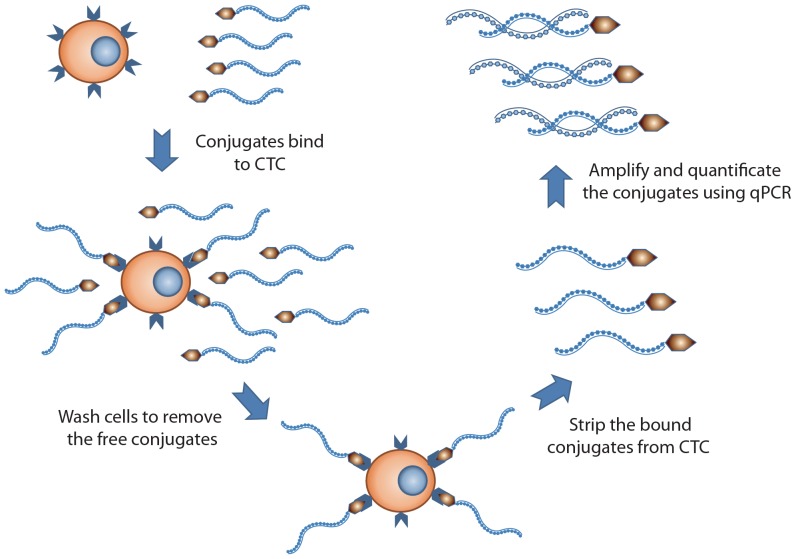
Outline of the ligand-targeted PCR detection for CTC. After negative enrichment by immunomagnetic depletion of leukocytes, CTCs were labeled with a conjugate of a tumor-specific ligand and an oligonucleotide. The labeled CTCs were washed thoroughly to remove free conjugates. Then the bound conjugates were stripped from CTC and analyzed by qPCR.

To advance CTC detection to the next level, there emerged various promising technologies for CTC enrichment: 1) CTC was positively, immunomagnetically captured by anti-EpCAM(epithelial cell adhesion molecule) antibody coated magnetic beads [7], [12], 2) CTC was enriched by immunomagnetic depletion of leukocytes with anti-CD45 antibody coated magnetic beads [5], [13]. 3) CTC was enriched by size-based filtration devices [14], 4) CTC was enriched by a chip-based device with signature microstructure [15], and 5) CTC was enriched by simultaneous depletion of erythrocytes and leucocytes using density gradient centrifugation [16], [17]. For subsequent, quantitative analysis of CTC, the enriched fraction using the aforementioned methods was immunofluorescently labeled and then examined microscopically or through flow cytometry [12], [18]–[21]. Though immunofluorescence based methods exhibited high specificity, the sensitivity may not be sufficient to detect rare cells at early stage of cancer [4], [12], [22]. Reverse transcriptase polymerase chain reaction (RT-PCR) was also used for CTC enumeration with high sensitivity [8], [23], [24]. However, post-transcriptional regulation that deregulates the gene expression was found in numerous cancer cells [25]–[27]. This regulation alters the gene expression through modifications of mRNA stability and/or transcriptional efficiency, and makes protein content not correlate with mRNA level. LT-PCR, developed by GenoSaber Biotech, showed promise in detecting CTC through surface proteins. Here we intend to evaluate the capability of the LT-PCR to examine CTC in NSCLC patients. In this method, CTC was labeled with a conjugate of a tumor-specific ligand folic acid, selectively bound to non-small cell lung cancer cells over-expressing folate receptor [28]–[31], and a synthesized oligonucleotide (Figure 1). The conjugate serves as an adapter molecule to convert a CTC into detector molecules “oligonucleotides” that can be amplified for quantitative analysis. This study is proposed to evaluate clinical value of detecting folate receptor positive CTC in NSCLC patients.

## Materials and Methods

### The reagents

The CytoploRare® circulating lung cancer cell kit was provided by GenoSaber Biotech Co. Ltd. (Shanghai, China). The kit comprises two components: one is for CTC enrichment and the other is for CTC detection and quantification. The enrichment component includes red cell lysis buffer, incubation buffer, anti-CD45 leukocyte depletion magnetic beads, washing buffer, labeling buffer, stripping buffer and neutralization buffer. The detection and quantification component includes PCR reaction buffer, primers, deionized water, positive and negative cell controls, PCR controls and standards. The primer sequences were listed as following. RT primer, 5′-CTCAACTGGTGTCGTGGAGTCGGCAATTCAGTTGAGGGTTCTAA-3′; Forward primer, 5′- TATGATTATGAGGCATGA-3′; Reverse primer, 5′-GGTGTCGTGGAGTCG-3′; Taqman Probe, 5′-FAM-CAGTTGAGGGTTC-MGB-3′.

Following the manufacturer's instruction manual, CTC was enriched by lysis of erythrocytes and immunomagnetic depletion of leukocytes from 3 mL blood sample. The enriched CTC was labeled with a conjugate of a tumor-specific ligand folic acid and a synthesized oligonucleotide. After labeling, the enriched CTC was washed thoroughly to remove the unbound conjugates. Then the bound conjugates were specifically stripped from the CTC surface and collected for quantitative PCR analysis (Figure 1). Before amplification, the conjugate first annealed and extended on the RT primer. After that the extended conjugate was amplified and analyzed using a Taqman probe based quantitative PCR method. In the above method, the circulating tumor cells were identified as folate receptor positive cells as labeled by folate-linked oligonucleotides.

### Cell line

We used human nasopharyngeal cancer cell line KB, which expresses folate receptor 1 (FR) on cell surface, to evaluate the recovery ratio in spiked cell assay. KB cells were cultured in folate-deficient RPMI-1640 medium (Life technologies) with 10% Fetal bovine serum (Life technologies) at 37°C in humidified atmosphere containing 5% CO_2_.

### Patients

Seventy-two patients with NSCLC, 20 with benign lung diseases, and 24 gender- and age-matched healthy donors were enrolled in the study. In the NSCLC patients, 33 patients were initially diagnosed, and 39 patients were on chemotherapy. Cancer patient characteristics are present in Table 1. The characteristics of patient cohort of benign diseases are listed in Table S1 in File S1. Three milliliters of peripheral blood were withdrawn into anticoagulant tubes containing EDTA from subjects participating this study. All patient and healthy volunteers included in this study signed written informed consent before donating blood. This study was approved by Hospital Ethics Committee of Shanghai Chest Hospital and Ethics Committee of Affiliated Hospital of Nantong University.

**Table 1:** Patient characteristics.

Characteristics		Initially diagnosed		On chemotherapy	
Category	Subcategory	No.	%	No.	%
Age,years	Median	58		59	
	Range	33–76		40–85	
Sex	Female	7	21	17	44
	Male	26	79	22	56
Tumor stage	I & II	10	30	6	15
	III	9	27	10	26
	IV	14	42	23	59
Histologic subtype	Adenocarcinoma	16	48	26	67
	SCC	11	33	7	18
	Others	6	18	6	15

Abbreviations: SCC, squamous cell carcinoma.

### Sample preparation

To prepare the blood samples for PCR analysis, CTCs were enriched by lysis of erythrocytes and subsequent depletion of leukocytes referring to the manufacturer's protocol. Briefly, the anticoagulant whole blood samples were first lysed by red cell lysis buffer (v∶v, 1∶4) for 15 min on ice. The cells were then treated with 200 µL anti-CD45 coated magnetic beads for 30 min to deplete leucocytes. After that, CTC was incubated with 10 µL labeling buffer (folate-linked oligonucleotide) for 40 min at room temperature. The cells were then washed 3 times with 1 mL wash buffer at 500 g. Finally, the cells were treated with 120 µL stripping buffer to remove the ligand-oligonucleotide conjugates. The supernatant were collected by centrifugation and neutralized by 24 µL neutralization buffer for further PCR analysis.

### PCR analysis

Real time quantitative polymerase chain reaction was performed using the CytoploRare® circulating lung cancer cell kit on ABI StepOne™ system (Life technologies). Two and half microliters of the prepared samples were added into a 25 µL PCR reaction system following the manufacturer's instruction manual. The PCR reaction conditions were as follows: denaturation at 95°C for 2 min, annealing at 40°C for 30 s, extension at 72°C for 30 s, then cooling at 8°C for 5 min; 40 cycles of denaturation at 95°C for 10 s, annealing at 35°C for 30 s, and extension at 72°C for 10 s. A serial of standards containing oligonucleotides (10^−14^ to 10^−9^ M, corresponding to 2 to 2×10^5^ CTC units/3 mL blood) are used for CTC quantification. All patient samples were tested in duplicates with 6 standards and 3 quality controls. Per the manufacturer's protocol, the mean intra-assay variance (the maximum difference between duplicates) should be <0.5 threshold cycle for the standards and quality controls, and <1 threshold cycle for tested samples.

### Recovery, linearity and limit of quantification studies

To establish a legitimate system to evaluate the accuracy and linearity of this assay, blood samples from healthy donors were pooled and aliquot into 3 mL samples, which were spiked with 5, 10, 20, 40, and 200 cultured KB cells. The unspiked, pooled blood served as a negative control (NC). Two hundred thousand cultured KB cells served as a positive reference (PR). The above experiments were repeated for 3 times. The observed cell counts were computed by the equation below:
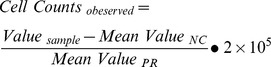
The recovery ratio was determined by dividing the quantity of the observed cell by the quantity of the spiked cells.

To determine the linearity of the assay, we have analyzed the observed and spiked CTC counts across all the data points by linear regression. We removed the confounding variable of percent recovery by using the observed value of the original sample divided by the dilution factors to determine the expected values for the dilution series for each tested sample.

### Immunostaining of enriched CTC

Three milliliter blood samples from two metastatic non-small cell lung cancer patients were enriched using the aforementioned protocol. The enriched CTC samples and cultured KB cells were fixed by methanol for 10 min at −20°C. Then the cells were labeled with a pan-cytokeratin monoclonal antibody conjugated to phycoerythrin (sc-8018PE, Santa Cruz, diluted at 1∶100) and a fluorescent conjugates of folic acid [19] (Wuxi AppTec, China, 10 nM) for 1 hour. After that, sample was washed three times with PBS, followed by mounting with 4′-6-diamidino-2-phenylindole (DAPI) containing mounting media (Life technologies), and subsequently subjected to image analysis using a confocal microscopy (Leica Microsystems, Germany).

### Clinical evaluations

To evaluate the clinical utility, we have conducted a double-blinded, two-center study on patients with benign and malignant lung diseases. In order to evaluate the difference among test groups, we used the student's t-test with Welch's correction for unequal variances with 95% confidence interval. To determine the threshold, specificity and sensitivity of the method, the data from patients with initially diagnosed NSCLC were subjected to the receiver operating characteristic (ROC) analysis by Prism (GraphPad software). The criterion to determine the optimal cutoff value in the ROC analysis is to maximize the total value of specificity and sensitivity.

## Results

### PCR calibration and CTC unit

As an external calibration curve, six standards containing a serial of concentrations of the conjugated oligonucleotides were used to calculate the quantity of folate receptors on CTCs (Figure S1). To extrapolate the quantity of CTC from the quantity of folate receptors, we use an arbitrarily defined unit, named CTC unit. As determined by the calibration curve, the quantity of folate receptor number on 1×10^5^ KB cells is 7.5×10^−14^ mol, which is defined as 1×10^5^ CTC units. The detectable range of the assay is from 2 to 2×10^5^ CTC unit. For quality controls, the intra-assay coefficient of variability (CV) is from 2.6% to 3.8%, and inter-assay CV is from 3.3% to 5.3% (Table S2 in File S1).

### Validation of the ligand-targeted PCR method for CTC quantification

**Figure 2:**
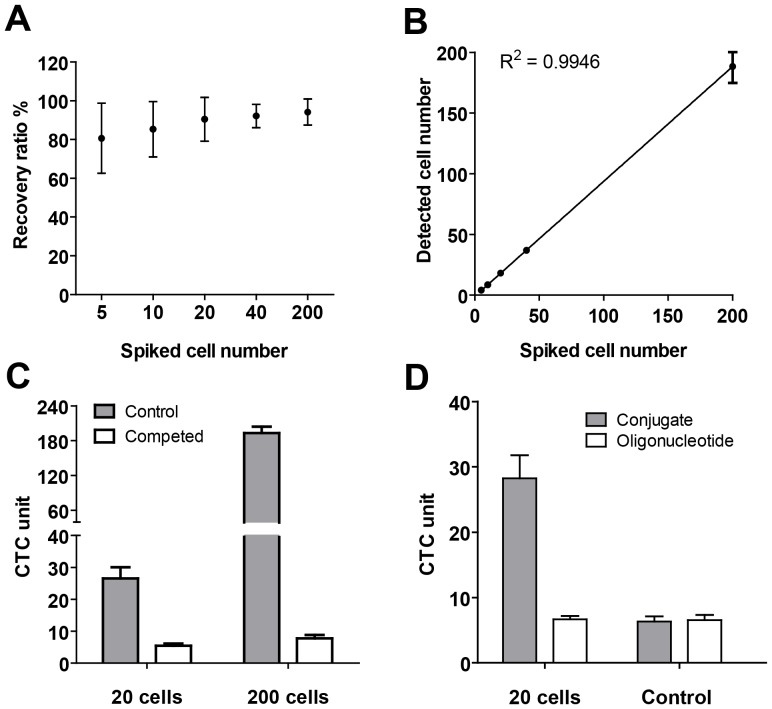
Validation of the ligand-targeted PCR method for CTC detection. A) Recovery ratio of spiked cell assay. B) Linear regression curve of spiked cell assay. C) Specificity test of the assay. Spiked KB cells were treated with (*competed*) or without (*control*) 10 µM folic acid before labeling. D) Healthy donors' blood samples spiked with 20 KB cells *(20 cells)* or without KB tumor cells *(control)* were labeled by 10 nM folate-oligonucleotide conjugate *(conjugate)* or unconjugated-oligonucleotide *(oligonucleotide)* after enrichment. Data are shown with Mean±SD from three independent assays.

The KB cell spiking study showed that >80% of the spiked cells could be recovered at each concentration (5, 10, 20, 40 and 200) (Figure 2A). The average CTC recovery ratio was 81% at 5 spiked cells in 3 mL blood, and even higher in samples spiked with higher concentration of cells. With the results from the spiking study, we performed the least squares fit for comparison of the observed and spiked quantity across all dilution series (Figure 2B). The regression equation was Y = 0.9455×−0.831 with R^2^ = 0.9946. The study indicated that over the tested CTC concentration the average recovery, as derived from regression analysis, is 94.6%.

To evaluate the intra-assay variance (within-run imprecision), we analyzed a sample of 100 KB cells in 3 mL blood in the same run in triplicate following the above protocol. Then we evaluated the inter-assay variance (between-run imprecision) by analyzing a same sample in triplicate in 6 separate assays on 6 different days. The intra-assay CV is 11.2%, and inter-assay CV is 14.2% (Table S3 in File S1).

To determine the specificity of this method we performed a competition study using 10 µM folic acid as a competing ligand to folate-linked oligonucleotide. The experiment results showed that 10 µM folic acid can block the conjugate from binding to the KB cells (Figure 2C). The blocking provides the proof of specificity of the folate-linked oligonucleotide bound to the folate receptor on spiked KB cells.

It should be noted that there are “background” signals for healthy donors' blood samples. To investigate the origin of the “background” signal, we labeled healthy donors' blood samples spiked with 20 KB cells or without KB tumor cells using 10 nM folate-oligonucleotide conjugate or unconjugated -oligonucleotide (Figure 2D). The blood samples spiked with no tumor cells bind to folate-linked oligonucleotide or unconjugated oligonucleotides indiscriminatively, as determined by qPCR. In contrast, the samples spiked with tumor cells can be obviously labeled by folate-conjugated oligonucleotide rather than the unconjugated oligonucleotide. The result suggested that “background” signals were caused by the binding of the oligonucleotides to the residual blood cells in the enriched samples. More importantly, the “background” signal caused by “oligo-binding effect” wouldn't compromise the specific detection of rare CTC as demonstrated below.

**Figure 3:**
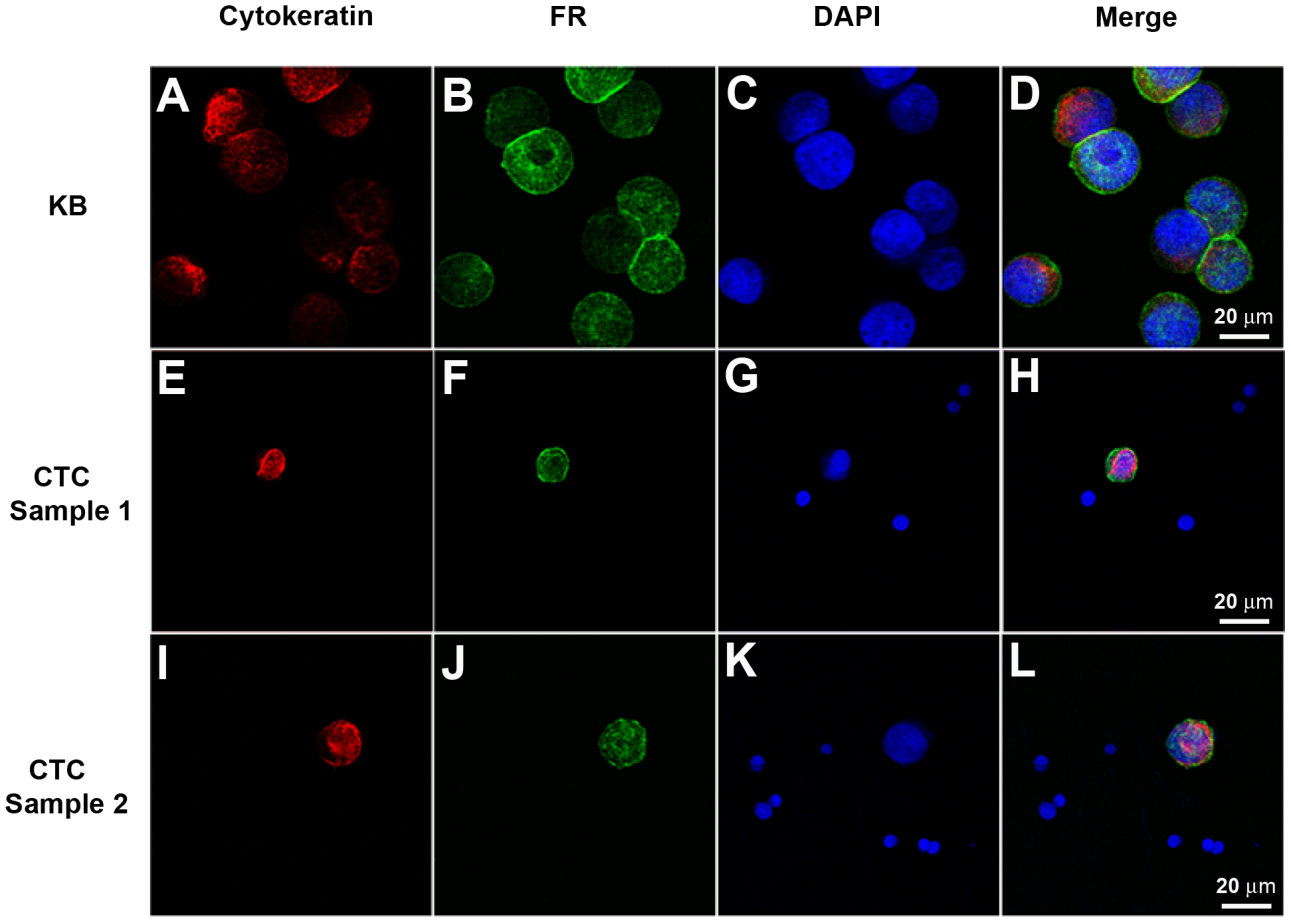
Immunostaining of the cultured KB cells and enriched CTCs. KB cells (A–D) and enriched CTCs from two metastatic NSCLC patients (CTC sample1: E–H; CTC sample 2: I–L) were fixed by methanol, and stained for cytokeratin (A,E and I) and folate receptor (FR) (B, F and J). The cell nucleus was labeled by DAPI (C, G and K). The merged images showed cytokeratin and folate receptor are expressed only in CTCs (H and L) and KB cells (D), not in haematologic cells.

To further evaluate the specificity of FR expression on CTC in NSCLC, three milliliter blood samples from two metastatic patients carrying FR positive CTC were enriched using the aforementioned protocol. Then the enriched CTC samples and cultured KB cells were fixed by methanol, and labeled by fluorescent conjugates of folic acid, monoclonal antibody for cytokeratins and nuclear staining reagent, DAPI. As shown in Figure 3, KB cells and the circulating tumor cells were specifically labeled by fluorescent folate conjugates (green) and cytokeratins antibody (red). In contrast, the small leukocytes can't be labeled by the fluorescent folate conjugates and cytokeratin antibody (Figure 3H and 3L). Further, under the microscope observation, the CTCs and cultured KB cells showed a similar expression level of folate receptor (Figure 3).

### CTC prevalence in clinical samples

As shown in Table 1, a total of 72 patients with NSCLC were enrolled in the study. As control groups, we obtained peripheral blood from 20 patients with benign lung diseases and 24 healthy donors.

**Figure 4:**
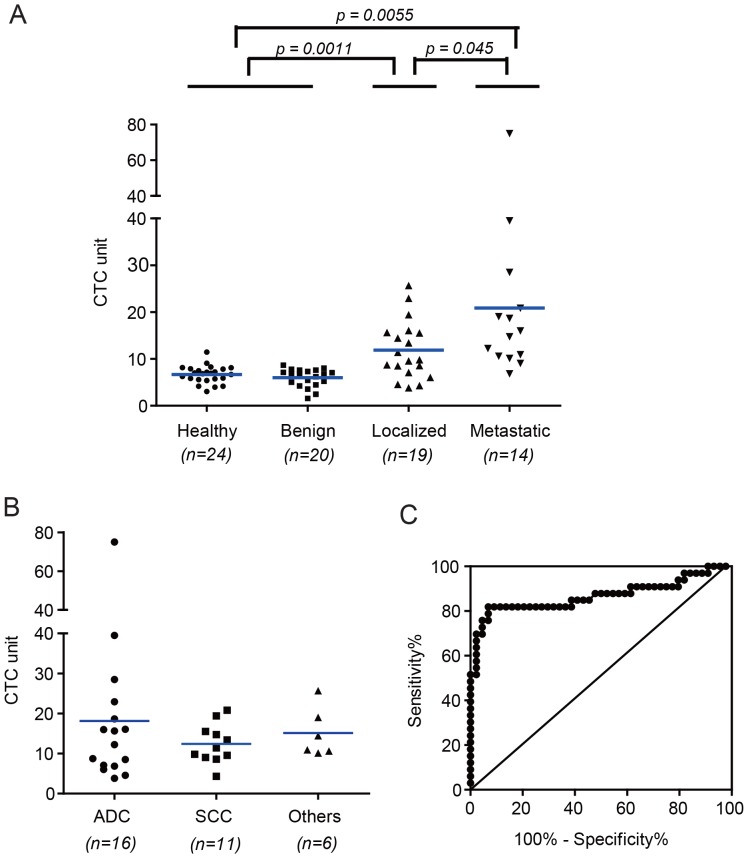
CTC prevalence in initially diagnosed patients. A) CTC levels in localized and metastatic malignant lung disease, benign lung disease and healthy donors. B) Distribution of CTC levels in different histological subtypes. ADC, adenocarcinoma; SCC, squamous cell carcinoma. C) Receiver operating characteristic (ROC) analysis between malignant lung disease group and benign disease and healthy group.

First, we compared CTC levels in cancer patients at initial diagnosis, benign disease patients, and healthy subjects. As shown in Figure 4A, the CTC levels in blood from patients with localized (stage I, II and III; mean 11.9 CTC unit; range 3.8–25.7 CTC unit; *p* = 0.0011) or metastatic (stage IV; mean 20.9 CTC unit; range 6.8–75.0 CTC unit; *p* = 0.0055) diseases is significantly higher than healthy volunteers (mean 6.7; range 3.0–11.4 CTC unit) and benign lung disease patients (mean 6.0; range 1.6–8.7 CTC unit). The metastatic patients have more CTC than localized patients (*p* = 0.045). The above results indicated that the localized and metastatic patients have distinct levels of CTC. In a more advanced analysis, we want to investigate the sensitivity of the method towards different histological subtypes of NSCLC. For the 16 adenocarcinoma patients, the CTC levels (mean 18.1, range 3.8–75.0) do not show significant difference from the 11 squamous cell carcinoma (SCC) patients (mean 12.4, range 4.3–20.8) and other histological subtypes (mean 15.1, range 10.1–25.7) (Figure 4B). This result suggested the expression of folate receptor might be similar across all histological subtypes in NSCLC patients.

To further analyze the sensitivity and specificity of the assay for different pathological stages and histological subtypes in NSCLC, we first need to establish the cut-off threshold by ROC analysis. Figure 4C showed that the cut-off threshold between control group (benign patients and healthy volunteers) and initially diagnosed cancer group was 8.5 CTC unit, the sensitivity is 81.8%, and the specificity is 93.2%. Obviously, CTCs were detected in blood samples from 82% (27 of 32) initially diagnosed NSCLC patients, whereas false positives in 20 benign disease patients (5%, 1 of 20) and 24 normal volunteers were negligible (8%, 2 of 24). As shown in Table 2, 80% (8 of 10) of stage I–II, 67% (6 of 9) of stage III, and 93% (13 of 14) of stage IV patients were found to have more than 8.5 CTC unit. In histological subtypes, 69% (11 of 16) of adenocarcinoma patients were CTC positive, and 91% (10 of 11) of SCC patients were CTC positive.

**Table 2:** Prevalence of CTC in initial diagnosed patients and association with clinical characteristics.

Characteristics		CTC unit > = 8.5		CTC unit <8.5	
Category	Subcategory	No.	%	No.	%
Lung cancer		27	82	6	18
Tumor stage	I & II	8	80	2	20
	III	6	67	3	33
	IV	13	93	1	7
Histologic subtype	Adenocarcinoma	11	69	5	31
	SCC	10	91	1	9
	Others	6	100	0	0
Benign		1	5	19	95
Healthy		2	8	22	92

Abbreviations: SCC, squamous cell carcinoma.

**Figure 5:**
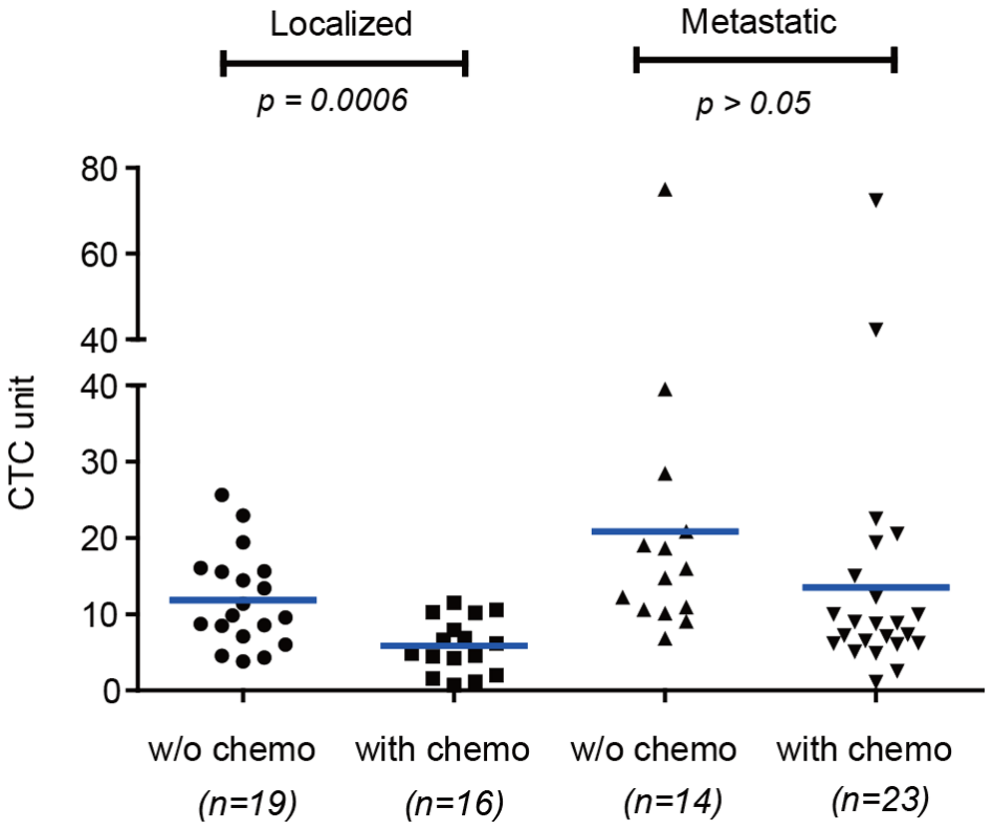
Correlation of CTC levels between NSCLC patients and chemotherapy treatment. *w/o chemo*, initially diagnosed NSCLC patients; *with chemo*, NSCLC patients on chemotherapy.

Published studies indicated the chemotherapy may compromise the presence of CTC in circulation [1], [4], [5]. We performed a preliminary study to compare the CTC number in NSCLC patients at initial diagnosis and on chemotherapy. As shown in Figure 5, 16 localized patients on chemotherapy (mean 5.8, range 0.7–11.5) have significantly lower CTC levels than the initially diagnosed patients (*p* = 0.0006); whereas the difference in CTC levels in metastatic patients with or without therapy was not so significant as the localized patients. Although the mean CTC level in metastatic patients at initial diagnosis was 20.9 CTC unit as compared to 13.5 CTC unit in patients on chemotherapy, the t-test did not verify that the difference was significant at a *p* value of 0.05. The statistical analysis suggested that the localized NSCLC patients may have better prognosis compared to the metastatic patients when put on chemotherapy.

## Discussion

We have evaluated LT-PCR technique for quantifying CTC in peripheral blood samples from NSCLC patients. The technology is based on negative depletion of leukocytes followed by labeling of CTC with folate-linked oligonucleotide and subsequent quantification with qPCR. Studies with healthy blood samples spiked with tumor cells demonstrate that the assay is specific, quantitative and linear from a range of 5–200 cells per 3 mL blood analyzed. Evaluation of patient blood samples demonstrates that the method is sensitive enough to stratify patients with different disease status (benign, localized and metastatic).

Potential advantages of LT-PCR to the oncologists may include 1) non-invasive assessment of tumor burden in peripheral blood, 2) ultrasensitive analysis that enables advanced stratification of lung diseases, 3) as low as 3 mL sampling volume that would not aggravate the patients' anemia caused by cytotoxic side effects, 4) real-time longitudinal monitoring of disease status and response to therapy, and 5) standardized platform that eliminates artificial analysis bias.

Most *in vitro* diagnostic blood tests for NSCLC cancer suffer from potential false negative and/or positive results. The use of anti-EpCAM coated magnetic beads was attempted for enrichment of CTC in NSCLC blood samples. However, clinical studies showed that low sensitivity of the EpCAM based enrichment in CTC detection for NSCLC patients [12], [22]. Published studies also indicated that non-EpCAM expressing cell may comprise the majority of CTC in NSCLC peripheral blood, and thus EpCAM based enrichment may fail to detect rare CTC [22]. We detected the folate receptor expression in EpCAM positive and negative CTCs in NSCLC patients. Anti-EpCAM coated magnetic beads were used to separate EpCAM-positive and EpCAM-negative fraction of CTCs. We found the FR positive CTC number was much higher in EpCAM-negative fraction than in EpCAM-positive fraction in all three NSCLC patients (Figure S2). The results indicated that EpCAM based capturing may miss large fraction of CTCs in lung cancer. Carcinoembryonic antigen (CEA), a glycoprotein involved in lung cancer development, is also found on normal or benign diseased cells released into circulation [32]. The applicability of CYFRA21-1, which is a cytokeratin 19 fragments released in the blood, has been investigated in studies in the diagnosis of NSCLC [33]–[35]. Although CYFRA21-1 may be the most sensitive tumor marker in advanced NSCLC, the sensitivity of the CYFRA21-1 is still low for early stage disease [33], [35].

Published data from tissue biopsy have shown 72%–83% lung cancer over-express the FR on cell surface [28]–[31]. Recent reports showed the FR positive CTC were found in ovarian cancers [19]. So far, no studies have been reported for the prevalence of FR positive CTC in NSCLC. In this study, we have investigated the clinical value of folate receptor positive CTC in NSCLC. Results indicated the FR is a potential surface marker in identifying CTC of NSCLC patients, even in early stage. Recent studies showed that, in primary tumor mass, FR expression is much lower in SCC than in adenocarcinoma in metastatic cancer [29]. However, we found the FR expression in CTCs is similar across all histological subtypes. This contradictory finding can be explained by different distribution of pathological stages for individual histological subtypes. Iwakiri *et al.* reported that the lower expression of folate receptor was found in the advanced stage compared to early stage in NSCLC [36]. In the patients enrolled in our study, 48% have adenocarcinomas (stage IV, 44%), 33% have squamous cell carcinomas (stage IV, 27%) and 18% have unspecified lung cancer. The lower FR positivity in adenocarcinomas in our study could be explained by the high percentage of late stage patients enrolled as compared to SCC group. As well, there exists another possibility that CTC may express FR different from primary tumor masses. Previous literature indicated differential gene expression between primary tumor and CTC sample [37]. A large-scale, well-designed clinical study would be needed to address the above concern.

Although folate receptor has been identified in normal tissues, including kidney, spleen and lung etc [28]. However, there are no cells expressing folate receptors in circulatory system except CTCs or activated monocytes [19], [38]. Preliminary studies found that this subpopulation of activated monocytes is barely detectable in the blood samples from healthy donors or benign patients [19], [39]. In 9% of the cases where “CTC” can be detected in healthy samples, there might exist possibility of illegitimate expression of monocytes. But the hypothesis needs to be verified in a further study. Folate receptor expression is also found in tumor activated macrophages [40]. In order to examine the macrophages effect in this detection system in NSCLC patients, anti-CD14 coated magnetic beads was used to deplete the activated macrophages in blood sample of NSCLC patients [41], after depletion of CD45-positive leukocytes. We found the CTC counts were barely changed under the “extra-CD14” treatment in NSCLC patients (Figure S3). This result suggested the depletion mechanism using anti-CD45 magnetic beads is sufficient to remove the impact of tumor-associated macrophages on CTC counts.

In this study, we found a low background “CTC” level in the healthy subjects and benign disease patients. As demonstrated from the specificity study, the background signal may result from the binding of oligonucleotide to the residual blood cells. Although previous literature described several mechanisms for the binding of the oligonucleotide to mammalian cells [42], there is no consensus for this biological process. However, these studies all demonstrated that the binding quantity of oligonucleotide is significantly lower compared to our conjugates. We are confident that the “background CTC” won't interfere with the detection of the real “CTC” in lung cancer patients.

Finally, because higher CTC counts were reported to associate with poor prognosis in metastatic cancers [2], [4], [43], LT-PCR would potentially identify the subgroup of patients with poor prognosis in early stage of disease and therefore aid oncologists to prescribe more effective therapies. In a more advanced analysis, the phenotype of CTC would provide direct evidence for oncologists to determine the optimal treatment regimen. With appropriate affinity ligand (e.g. antibody, peptide, small chemical molecules, etc.), the LT-PCR can be further explored for phenotyping rare CTC in a multiplex, high-throughput fashion.

## Supporting Information

Figure S1 Calibration curve of qPCR and lineality parameters.
(TIF)

Figure S2 Folate receptor expression in EpCAM positive and negative CTCs in NSCLC patients. CTC samples were enriched from 3 mL blood of three FR positive NSCLC patients by lysis of erythrocytes and immunomagnetic depletion of leukocytes. Then the enriched CTC samples were incubated with anti-EpCAM magnetic beads (Life technologies, Cat No. 16203) for 30 min. After immunomagnetic isolation for 10 min, the EpCAM positive cells were captured by the magnetic beads. The EpCAM negative cells were collected from the supernatant by centrifuging at 600 g for 15 min. After that, the two fractions of CTC sample were labeled and detected as the aforementioned protocol. Data are shown with Mean±SD from three independent qPCR assays.
(TIF)

Figure S3 The impact of tumor-associated macrophages in FR positive NSCLC patients. CTC samples were enriched from 3 mL blood of three FR positive NSCLC patients by lysis of erythrocytes and immunomagnetic depletion of CD45-positive leukocytes. Then the enriched CTC samples were incubated with anti-CD14 magnetic beads (Life technologies, Cat No. 11119D) (*CD14 beads*) or IgG-conjugated beads (Life technologies, Cat No. 11201D) (*Control*) for 30 min. After immunomagnetic depletion CTC samples were labeled and detected as the aforementioned protocol. Data are shown with Mean±SD from three independent qPCR assays.
(TIF)

File S1 contains all supporting tables (**Table S1**, **Table S2** and **Table S3**) in the manuscript. **Table S1.** Characteristics of Benign Disease Patients. **Table S2.** Precision of the PCR amplification assay for quality controls. **Table S3.** Precision of the LT-PCR assay for spiked cells and quality controls.
(PDF)
