# Supplementary figures and images for: Quantification of Rare Circulating Tumor Cells in Non-Small Cell Lung Cancer by Ligand-Targeted PCR
Source: PLoS One. 2013 Dec 6;8(12):e80458. doi: 10.1371/journal.pone.0080458 (PMC3855610; doi:10.1371/journal.pone.0080458)

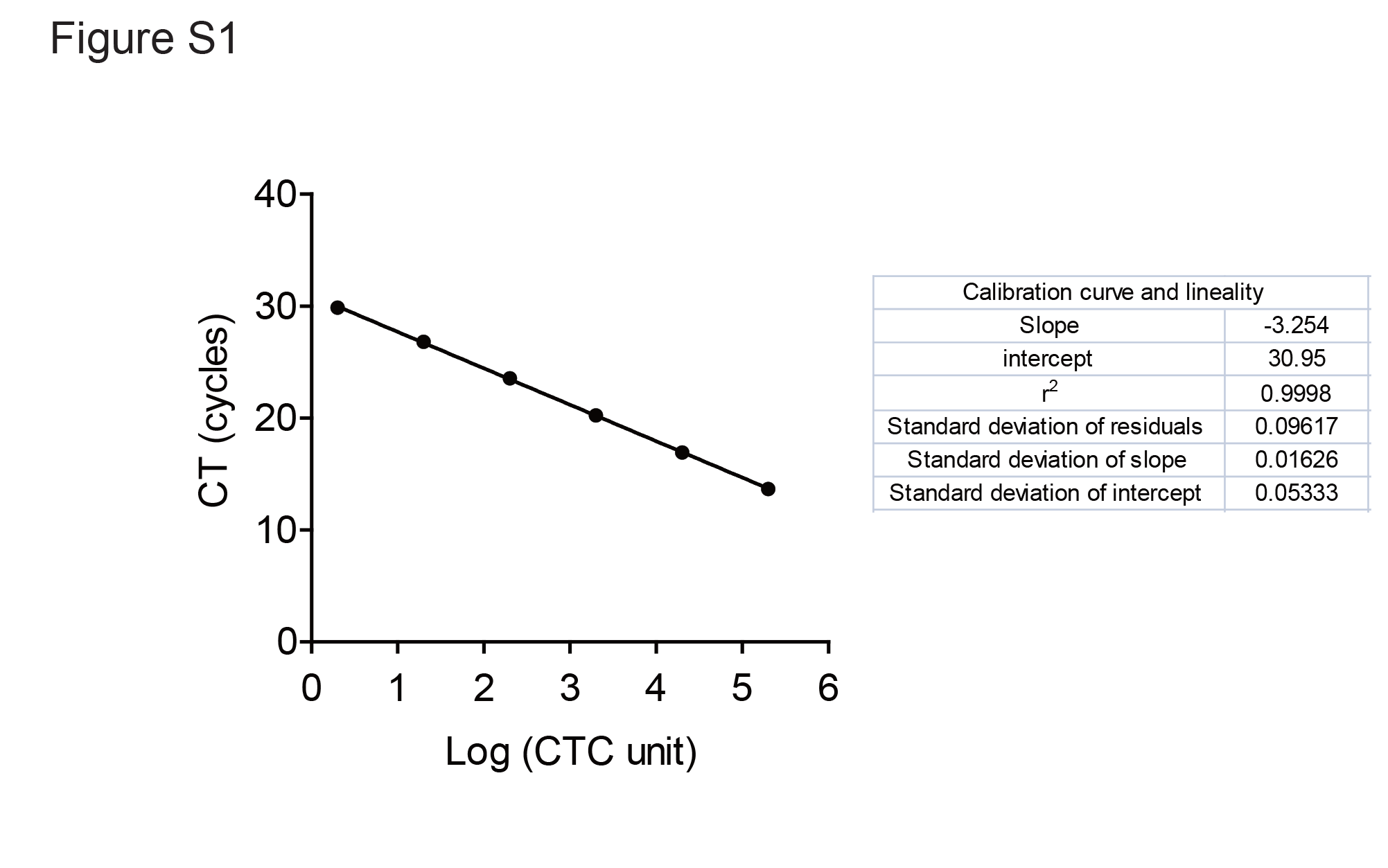

Supplement: Figure S1 — Calibration curve of qPCR and lineality parameters. (TIF) [file pone.0080458.s001.tif]

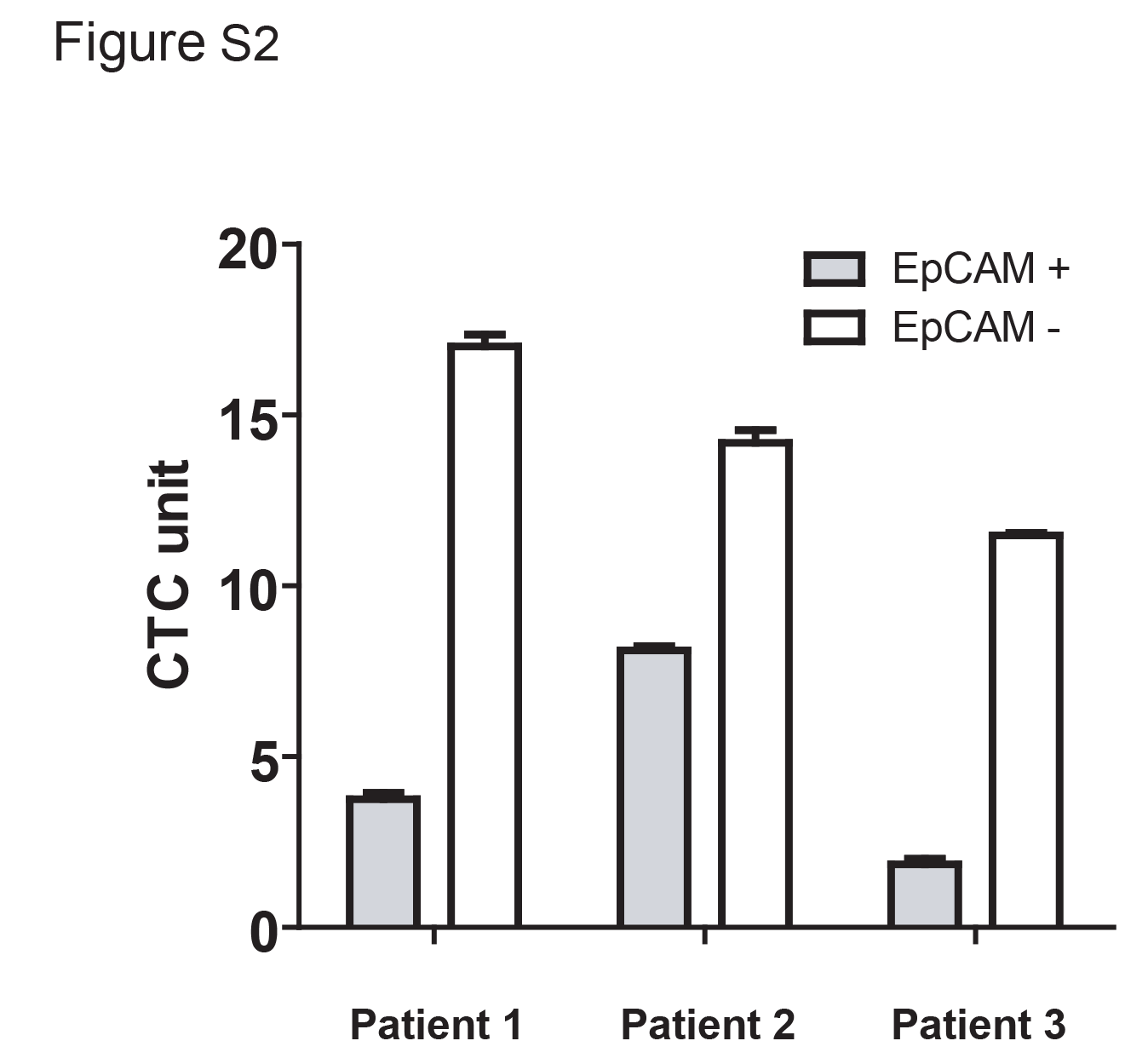

Supplement: Figure S2 — Folate receptor expression in EpCAM positive and negative CTCs in NSCLC patients. CTC samples were enriched from 3 mL blood of three FR positive NSCLC patients by lysis of erythrocytes and immunomagnetic depletion of leukocytes. Then the enriched CTC samples were incubated with anti-EpCAM magnetic beads (Life technologies, Cat No. 16203) for 30 min. After immunomagnetic isolation for 10 min, the EpCAM positive cells were captured by the magnetic beads. The EpCAM negative cells were collected from the supernatant by centrifuging at 600 g for 15 min. After that, the two fractions of CTC sample were labeled and detected as the aforementioned protocol. Data are shown with Mean±SD from three independent qPCR assays. (TIF) [file pone.0080458.s002.tif]

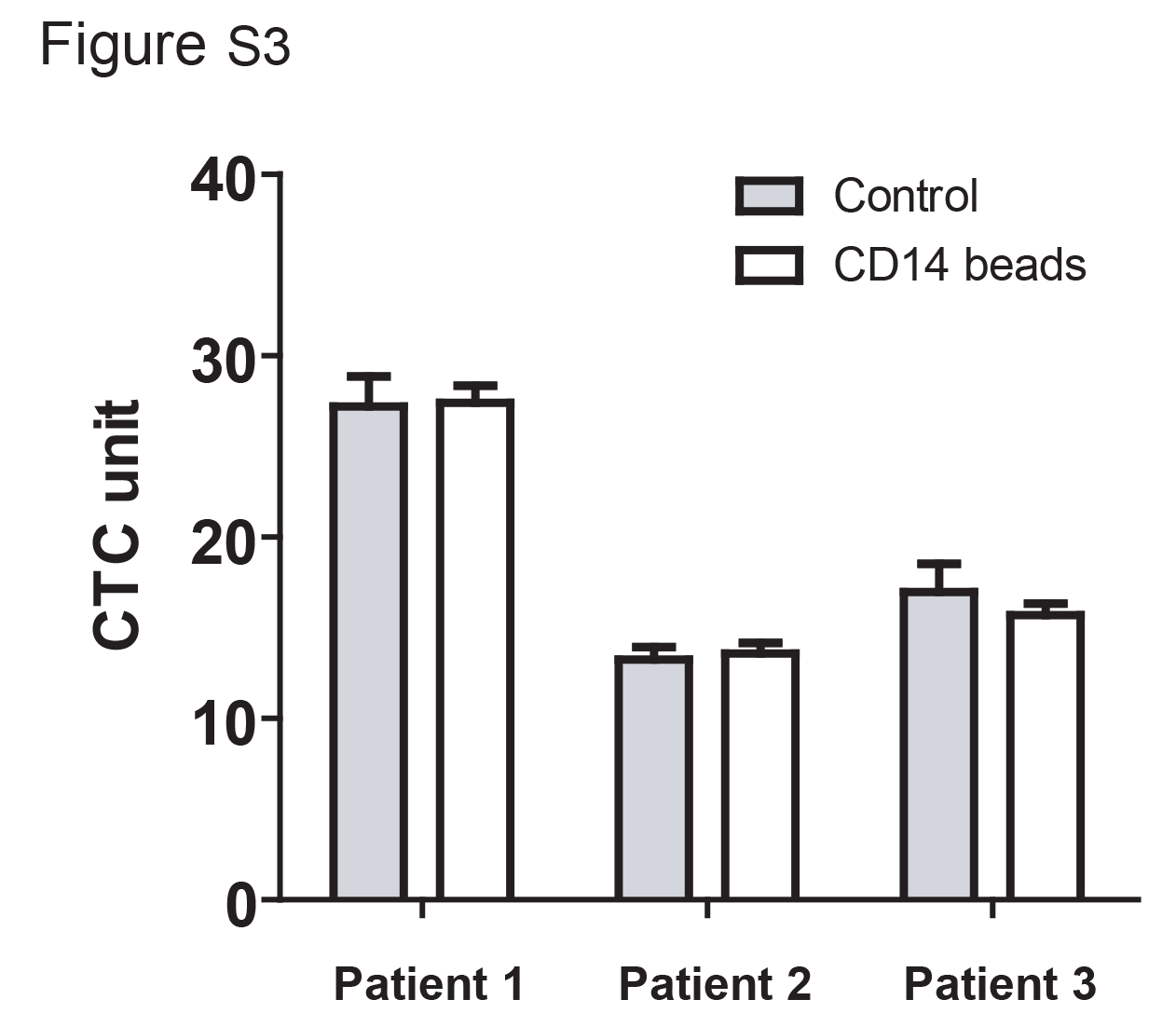

Supplement: Figure S3 — The impact of tumor-associated macrophages in FR positive NSCLC patients. CTC samples were enriched from 3 mL blood of three FR positive NSCLC patients by lysis of erythrocytes and immunomagnetic depletion of CD45-positive leukocytes. Then the enriched CTC samples were incubated with anti-CD14 magnetic beads (Life technologies, Cat No. 11119D) (CD14 beads) or IgG-conjugated beads (Life technologies, Cat No. 11201D) (Control) for 30 min. After immunomagnetic depletion CTC samples were labeled and detected as the aforementioned protocol. Data are shown with Mean±SD from three independent qPCR assays. (TIF) [file pone.0080458.s003.tif]
