# Supplementary material for: Quantification of Rare Circulating Tumor Cells in Non-Small Cell Lung Cancer by Ligand-Targeted PCR
Source: PLoS One. 2013 Dec 6;8(12):e80458. doi: 10.1371/journal.pone.0080458 (PMC3855610; doi:10.1371/journal.pone.0080458)
Supplement: File S1 — contains all supporting tables (Table S1, Table S2 and Table S3) in the manuscript. Table S1. Characteristics of Benign Disease Patients. Table S2. Precision of the PCR amplification assay for quality controls. Table S3. Precision of the LT-PCR assay for spiked cells and quality controls. (PDF) [file pone.0080458.s004.pdf]

File S1

Table S1. Characteristics of Benign Disease Patients

**Characteristics of Benign Disease Patient**		
**Characteristics**	**No.**	**%**
Age, years		
median	56	
Range	18-83	
Sex		
Female	9	45
Male	11	55
Diseases		
**Bacterial pneumonia**	7	35
**Chronic bronchitis**	9	45
**Pulmonary tuberculosis**	1	5
**Others**	3	15

Table S2. Precision of the PCR amplification assay for quality controls.

**Precision of the PCR amplification assay for quality controls**			
	QC1	QC2	QC3
**Intraassay precision (n=3), CTC unit**			
**Sample**			
**1**	63.2	6.6	U.D.
**2**	62.3	6.8	U.D.
**3**	65.5	6.3	U.D.
**Mean**	63.7	6.6	/
**SD**	1.7	0.3	/
**CV %**	2.6	3.8	/
**Interassay precision (n=6), CTC unit**			
**Day**			
**1**	61.8	5.9	U.D.
**2**	65.7	6.8	U.D.
**3**	62.4	6.2	U.D.
**4**	67.2	6.7	U.D.
**5**	63.3	6.2	U.D.
**6**	61.5	6.0	U.D.
**Mean**	63.7	6.3	/

**SD**	2.1	0.3	/
**CV %**	3.3	5.3	/

Based on the manufacture’s protocol, the concentrations ranges of QC1 and QC2, are 20.0-30.0 and 2.0-3.0 CTC unit/μL respectively. QC3 is a negative control. In the assay, we used 2.5 μL quality control samples for qPCR analysis, data showed the all measured values meet the criteria of quality control.
Abbreviations: U.D., Under the limit of detection.

Table S3. Precision of the LT-PCR assay for spiked cells and quality controls

**Precision of the LT-PCR assay for spiked cells and quality controls**			
	20 KB cells in 3 mL blood	CTC-QC1	CTC-QC2
**Intraassay precision (n=3), CTC unit**			
**Sample**			
**1**	16.8	184.7	<1
**2**	24.4	212.2	<1
**3**	28.3	246.4	<1
**Mean**	25.2	221.1	/
**SD**	2.8	22.2	/
**CV %**	11.2	10.1	/
**Interassay precision (n=6), CTC unit**			
**Day**			
**1**	25.4	180.4	<1
**2**	26.2	240.3	<1
**3**	18.3	234.4	<1
**4**	19.8	220.3	<1
**5**	22.4	178.8	<1
**6**	24.2	230.6	<1
**Mean**	22.1	209.9	/
**SD**	3.2	27.5	/
**CV %**	14.2	13.1	/

To evaluate the precision of LT-PCR assay, 20 spiked KB cells in 3 mL blood from healthy donors were tested with quality controls (CTC-QC1 and CTC-QC2), following the manufacture’s protocol. CTC-QC1 is a positive control to evaluate the efficiency of the conjugate labeling, whereas CTC-QC2 is a negative control to

monitor unbound conjugates in the prepared samples. Data showed all measured values meet the quality control criteria.
